# Pneumomediastinum and Pericardium During Labour: A Report on a Rare Postpartum Phenomenon

**DOI:** 10.7759/cureus.50850

**Published:** 2023-12-20

**Authors:** Nelson Chen, Tessa K Daly, Roshini Nadaraja

**Affiliations:** 1 General Surgery, Austin/Northern Hospital, Melbourne, AUS; 2 General Surgery, Northern Hospital, Melbourne, AUS

**Keywords:** labour and delivery, subcutaneous emphysema, pneumopericardium, spontaneous pneumomediastinum (spm), postpartum pneumomediastinum, hamman’s syndrome

## Abstract

Hamman’s syndrome (HS) is characterised by spontaneous pneumomediastinum and subcutaneous emphysema. It is a rare phenomenon that can occur during labour. Its incidence is 1 in 100,000 births and predominantly affects young primiparous women with prolonged labour. Patients commonly present with subcutaneous emphysema, chest pain, and dyspnoea.

We present the case of a 20-year-old primigravida female with no other medical history who had prolonged labour at 43 weeks gestation. Sudden-onset, right-sided cheek pain and swelling was noted immediately after delivery accompanied by pleuritic chest pain. Chest X-ray (CXR) and computed tomography (CT) demonstrated significant pneumomediastinum and pneumopericardium with subcutaneous emphysema extending to the neck. She was managed symptomatically in addition to antibiotics and discharged after three days with complete resolution of symptoms. No concerns were raised during the follow-up.

HS is a rare phenomenon that can occur during labour, particularly in young primiparous females with a prolonged second stage. Radiological investigations in the form of CXR and CT are recommended to rule out life-threatening complications and other conditions that may require immediate management. HS occurs due to rupture of peripheral alveoli secondary to increased intrathoracic pressures from excessive Valsalva manoeuvre allowing air to dissect and enter into the mediastinum.

Pneumopericardium in association with HS is extremely rare. It is particularly clinically important because it can cause cardiac tamponade requiring immediate surgical management. HS is otherwise a self-limiting condition and management is symptomatic only.

Our case is unique due to the presence of pneumopericardium in association with HS, the fourth ever reported in the literature. Due to its rarity, the incidence of tamponade in this cohort of patients is yet to be delineated.

## Introduction

Spontaneous pneumomediastinum (SPM) during labour is a very rare phenomenon. Hamman’s syndrome (HS) is a constellation of clinical signs characterised by SPM and subcutaneous emphysema that was first described by Louis Hamman in 1945 [1]. The incidence of postpartum HS is approximately 1 in 100,000 births [2] and predominantly affects young primiparous women during the second stage of labour in a vaginal delivery [2,3]. HS is thought to be caused by repetitive hyperinflation of the lungs, barotrauma, and high intra-alveolar pressures due to excessive Valsava manoeuvre. This can result in alveolar rupture and air dissection into the mediastinum, hila, and subcutaneous tissues, a phenomenon known as the Macklin effect [2,4-6].

Patients commonly present with chest pain, subcutaneous emphysema, and dyspnoea [2]. The diagnosis of SPM is made clinically but chest imaging, including X-rays and computed tomography (CT), is required to exclude other life-threatening conditions such as tension pneumothorax, Boerhaave’s syndrome, pulmonary embolus, and myocardial infarction [2,5,7] which may necessitate urgent treatment.

HS is generally a self-limiting condition requiring only conservative symptomatic treatment [7]. There is currently no established evidence-based treatment regime [7]. However, due to the significant radiographic findings and potential complications, clinicians should be cognisant of this phenomenon.

Pneumopericardium in the setting of postpartum HS is particularly rare [2] and should be monitored as it may infrequently progress to tamponade which requires immediate surgical intervention [8]. A systematic review by La Verde et al. suggested that because of the low prevalence of postpartum SPM, there is a lack of data in the literature to guide treatment [2]. Only three cases of pneumopericardium were identified in their systematic review which is the most extensive to date [2]. We present the fourth-ever case report of significant pneumopericardium in HS in an otherwise well primigravida patient.

## Case presentation

A 20-year-old primigravida female was admitted to hospital 43 weeks pregnant in the first stage of labour. She had no past medical history, no regular medications, and was a non-smoker. Her pregnancy was otherwise uneventful with attendance of all antenatal care. Delivery was complicated by a prolonged period of active labour requiring an epidural for pain control involving significant strain and effort of pushing. Spontaneous vaginal delivery was achieved after 12 hours of active labour in the left lateral decubitus position complicated by a second-degree vaginal tear and postpartum haemorrhage with an estimated blood loss of 1 L.

Immediately following delivery, the patient complained of sudden-onset, right-sided cheek pain and swelling. This was accompanied by ipsilateral pleuritic chest pain. She did not have dyspnoea, dysphonia, dysphagia, palpitations, nausea, or vomiting nor was there any preceding coryzal symptoms, sick contacts, or any recent air travel.

Examination demonstrated a swollen right face and neck. She was tachycardic with a heart rate of 105 beats/minute but her remaining vital signs were normal and she remained afebrile throughout. Her trachea was midline with palpable non-tender crepitus over the right cheek, neck, and superior portion of her anterior chest wall. Auscultation demonstrated equal air entry bilaterally with no added sounds. Heart sounds were unremarkable. There were no audible precordial crepitations which if present constitute Hamman’s sign.

**Figure 1:**
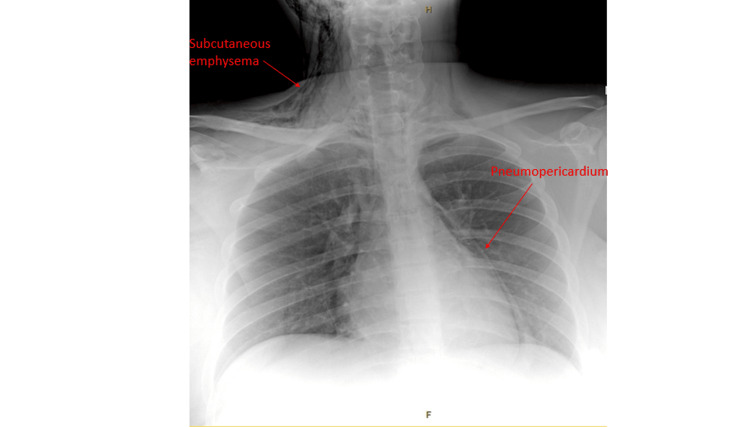
Anteroposterior view of chest X-ray demonstrating right pneumopericardium, pneumomediastinum, and subcutaneous emphysema along both sides of the neck.

An electrocardiography revealed a normal sinus rhythm with no segmental changes or arrhythmias. Biochemical markers including a troponin level were all within the normal range. An urgent chest X-ray (CXR) (Figure 1) revealed a large pneumopericardium and pneumomediastinum as well as extensive subcutaneous emphysema involving the right side of the neck and face.

**Figure 2:**
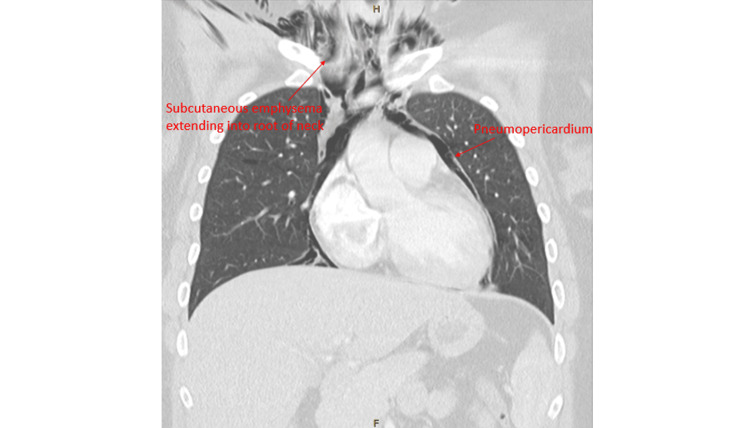
Coronal view of CT chest demonstrating significant pneumopericardium, pneumomediastinum, and subcutaneous emphysema extending to the root of the neck.

**Figure 3:**
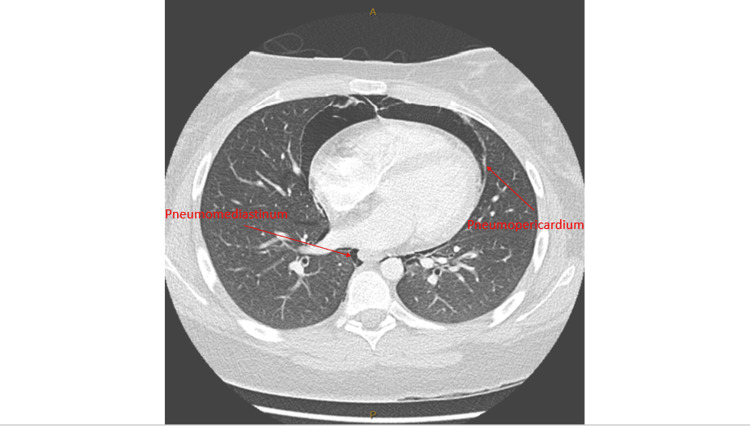
Axial view of CT chest demonstrating pneumomediastinum and pneumopericardium.

A subsequent CT of the chest with intravenous contrast (Figures 2, 3) demonstrated significant pneumomediastinum extending to the root of the neck, a moderate pneumopericardium, and right-sided subcutaneous emphysema indicating a diagnosis of HS. No pneumothorax, pulmonary embolism, or site of rupture/disruption was identified.

Tissue swelling increased over the next 12 hours with extension into the right side of the neck resulting in moderate stiffness. She did not develop any subsequent dysphonia, dysphagia, dyspnoea, airway compromise, fevers, palpitations, fevers, or chills.

The patient remained in the hospital for three days for close monitoring and was treated conservatively. She was managed with analgesia and a normal diet, as well as intravenous antibiotics in the form of co-amoxiclav to cover for potential mediastinitis. A repeat CXR the following day showed no progression of the pneumopericardium or pneumomediastinum. She was discharged three days later following the complete resolution of her symptoms and was followed up in the surgical outpatient clinic with no reported concerns.

## Discussion

SPM otherwise known as mediastinal emphysema is defined by air within the mediastinum without an obvious cause such as viscous perforation, infection, or trauma [8]. SPM in the setting of labour was first reported by Simmons in 1784 [9], but the combination of SPM and subcutaneous emphysema, known collectively as HS, was only first reported by Hamman in 1945 [1]. Overall, 70% of non-obstetric cases occur in males in their 20s [10], with risk factors including vomiting, labour, physical exercise, coughing, and convulsions, as well as those with chronic lung diseases, smokers, or those in labour [4]. The incidence of SPM in pregnancy is rare, occurring in approximately 1 in 100,000 vaginal deliveries [11]. Most cases of HS that occur are young primiparous females with a prolonged second stage. This was confirmed in La Verde’s systematic review in 2022 which demonstrated 76.25% of patients were primiparous with a median age of 24 [2]. Further, 55% of patients developed SPM during the second stage of labour, followed by 16.25% after delivery (fourth stage) [2]. Dudley et al. suggested that the risk of HS is also increased in pregnancies associated with macrosomia [12].

Swelling and subcutaneous emphysema are the most common presenting symptoms of HS, occurring in 91% of patients in La Verde’s largest systematic review of HS [2]. Other notable symptoms include chest pain, dyspnoea, crepitus, hoarse voice, and tachycardia. Less common symptoms include vomiting, coughing, odynophagia, neck pain, hearing loss, and haemoptysis [2]. Hamman’s sign is defined as crackling or crepitus synchronised with heart sounds heard on prechordial auscultation, and may also be present in the setting of mediastinal emphysema [6]. This is best elicited in the left lateral decubitus position during systole [13].

HS itself is a benign and self-limiting phenomenon but other serious life-threatening complications of pregnancy and labour need to be considered, including pulmonary embolus, myocardial infarction, tension pneumothorax, aortic dissection amniotic fluid embolism, mediastinitis, and spontaneous oesophageal rupture as these require immediate treatment [2,3,5,6,14]. For this reason, investigations such as a CXR are considered an important initial radiological modality in the diagnosis of HS [15,16]. CT chest with IV contrast, on the other hand, has better resolution and can detect smaller pockets of trapped air while also ruling out other life-threatening diagnoses [2,4,17]. A CT chest can also differentiate between pneumomediastinum and pneumopericardium which is reportedly less favourable and may require further treatment [8] if tamponade occurs [2,4]. Diagnosis of HS is, therefore, a combination of history, clinical signs, and radiological evidence excluding other complications [2,4].

The pathophysiology of HS is suggested to be due to increased alveolar pressure causing rupture of peripheral alveoli resulting in pneumomediastinum and subcutaneous emphysema [16,18,19]. This can occur in the setting of excessive Valsalva manoeuvre during the second stage of labour in normal vaginal delivery [16,20]. It can also occur with excessive retching, screaming, coughing, and straining [6,15,21]. The increased intrathoracic pressure creates a pressure gradient into the perivascular connective tissue, thereby allowing the air to dissect and enter the mediastinum, which is known as the Macklin effect [6,15,22]. The air then continues to shear along fascial planes within the subcutaneous and retroperitoneal tissues [3]. If the air enters the pleural space, a concomitant pneumothorax may be present [3].

HS is a benign self-limiting condition that resolves within two weeks with bed rest and conservative symptomatic management comprising oxygen, bronchodilators, analgesia, and anxiolytics [2,4,7,16]. La Verde et al. found that 50% of patients were managed with observation only or conservative/symptomatic treatment [2]. Antibiotics can also be administered if there is a suspicion or concern for the development of mediastinitis [2,23]. However, in the case of a pneumothorax, cardiorespiratory collapse can occur which may require urgent treatment [15]. Tension pneumomediastinum is a potentially life-threatening complication where high mediastinal pressures can result in dyspnoea, cyanosis, jugular venous distension, hypotension, and shock [16]. This requires urgent surgical intervention with splitting of the sternum to allow the trapped air to escape as it will lead to circulatory collapse and death if left untreated [15,16]. Mediastinitis is another potential complication with a high mortality rate; however, it is more commonly associated with oesophageal perforation (Boerhaave’s syndrome) rather than distal alveolar rupture [16]. Other extremely rare complications reported by La Verde et al. include pharyngeal rupture, hydropneumothorax, Takotsubo cardiomyopathy, and oesophageal rupture [2].

Recurrence in subsequent pregnancies is uncommon, and, currently, there are no evidence-based management guidelines in these scenarios [3,4,15,16]. Some studies have suggested measures for the prevention of HS including the use of early epidural analgesia to prevent active pushing, avoidance of nitrous oxide which increases intra-alveolar pressure, and early use of forceps to decrease the duration of the second stage of labour [3,24,25]. In cases where HS is recognised early, it is suggested that the delivery be hastened to minimise the progression of the mediastinal emphysema and barotrauma from excessive intra-alveolar pressures [25]. Oshovskyy et al. suggest it is not unreasonable to formulate a delivery plan at 37 weeks for those with a history of HS as the incidence of recurrence is unknown [26]. Patients who have recurrent HS should undergo further investigations to rule out pulmonary or oesophageal pathology [8].

## Conclusions

HS is a rare benign condition that can occur at any stage of labour but most frequently in the second stage. It is most common in young primiparous women who have had prolonged and difficult labour. CXR and chest CTs are the main imaging modalities used to assess the degree of pneumomediastinum/pneumopericardium and the associated complications, as well as to rule out sinister causes of chest pain that might require immediate treatment.

Pneumopericardium is an extremely rare finding in labour-related HS. In the case of rapid deterioration, tamponade should be suspected and managed appropriately. However, because of its rarity, the incidence of this is not known. HS is otherwise a self-limiting condition and management is largely conservative and symptomatic.
